# Digital health literacy as a predictor of patient safety culture: evidence from a multi-centre study of Jordanian nurses

**DOI:** 10.1080/20523211.2026.2650578

**Published:** 2026-04-13

**Authors:** Nadia Al Mazrouei, Khalid Awad Al-Kubaisi, Derar H. Abdel-Qader, Abduelmula R. Abduelkarem, Karem H. Alzoubi, Moh'D. Ahmad Shara, Richard Mottershead

**Affiliations:** a Department of Pharmacy Practice and Pharmacotherapeutics, College of Pharmacy, University of Sharjah, Sharjah, United Arab Emirates; b Faculty of Pharmacy and Medical Science, University of Petra, Amman, Jordan; c Department of Pharmaceutical Science, College of Pharmacy, QU Health, Qatar University, Doha, Qatar; d Sakina Behavioural Science Institute, SEHA, Al Ain, United Arab Emirates

**Keywords:** Digital health literacy, patient safety culture, nursing, Jordan, electronic health records, cross-sectional study, nursing informatics

## Abstract

**Background:**

The digitalisation of healthcare requires nurses to possess strong Digital Health Literacy (DHL) to ensure patient safety. This study aimed to evaluate the relationship between DHL and perceptions of PSC among registered nurses in Jordan and to identify the demographic and professional factors associated with varying levels of DHL.

**Methods:**

A multi-center, cross-sectional survey was conducted from July to September 2025 across five public, private, and university-affiliated hospitals in Jordan. A sample of 500 registered nurses was recruited using stratified random sampling. Data were collected using a validated, seven-part composite questionnaire. Data were analyzed using descriptive statistics, reliability analysis (Cronbach’s alpha), and structural equation modelling.

**Results:**

The results confirmed the study's primary hypothesis, revealing a significant and strong positive predictive relationship between DHL and PSC (*β* = 0.42, *p* < 0.001), with DHL explaining 28.1% of the variance in PSC. Nurses reported moderately high DHL overall (*M* = 3.28), with strengths in operational skills but lower confidence in evaluating information reliability. Perceptions of patient safety culture were positive (*M* = 3.71), with Teamwork Climate rated highest (*M* = 4.21) and Perceptions of Management lowest (*M* = 3.34). Significant disparities in DHL were found; higher levels of education (*p* < .001) and working in a university-affiliated hospital (*p* = 0.001) were associated with higher competency. Notably, no significant differences were observed based on age or years of experience. Lack of adequate training and insufficient time were identified as the primary organisational barriers to technology use.

**Conclusion:**

Digital health literacy was significantly associated with better patient safety culture among Jordanian nurses, driven more by organisational and educational factors than generational stereotypes. To realise the safety benefits of digital health investments, institutions should strengthen nurses’ digital and critical appraisal skills and remove organisational barriers.

## Background

The global healthcare landscape is undergoing a profound digital transformation, as the implementation of national Electronic Health Records (EHRs), telehealth infrastructures, and AI-driven decision-support systems changes the competencies required for contemporary nursing practice (Kinnunen et al., 2022; Malak et al., 2022; Strudwick, 2023; Topaz & Pruinelli, 2017). This evolution necessitates a shift in nursing skills to balance technological integration with the core values of patient-centred care (Alnasser et al., 2024). Central to this new paradigm is Digital Health Literacy (DHL), the ability to effectively find, appraise, and apply digital health information. This skill set is now widely regarded as a fundamental requirement for delivering safe, efficient, and evidence-based care (Corvo et al., 2024). As the largest professional group in the clinical workforce, nurses are at the epicentre of this transformation, with national health initiatives in countries, such as Jordan emphasising the critical role of staff in technology uptake (Abed et al., 2022). Within this study, DHL is conceptualised not just as basic operational EHR competency, but also encompasses the higher-order information appraisal skills critical for evaluating the reliability and clinical relevance of digital health data.

Research increasingly indicates that a digitally literate nursing workforce can directly support the delivery of safe and effective care, leading to improved patient outcomes (Fitzpatrick, 2023; Macalindin et al., 2024). However, the integration of digital tools is not without its challenges. The increased administrative workload associated with EHRs can lead to information overload and contribute to nurse burnout, while poorly designed systems can disrupt established nursing workflows (Kuek & Hakkennes, 2020; Thomas, 2023). This ‘information anxiety' can negatively impact nurses’ core competencies and their ability to make sound clinical decisions (Zhao et al., 2024). A systematic review of nursing processes in digital systems highlighted that while data collection (assessment) is well-supported, the documentation of nursing interventions and outcome evaluations remains inadequate, potentially hindering clinical reasoning and obscuring the value of nursing care (Hants et al., 2023; Shudayfat et al., 2023).

In the Jordanian context, research into nurses’ DHL has revealed a mixed but generally positive picture. A cross-sectional study found that Jordanian nurses achieved very desirable results in operational and navigational skills (Hants et al., 2023). However, this descriptive study did not investigate how DHL might influence critical outcomes, such as the collective Patient Safety Culture (PSC), the shared values, beliefs, and norms that determine how patient safety is managed and prioritised within a clinical unit (Sexton et al., 2006). This represents a critical gap, as a highly sophisticated digital infrastructure may fail to improve safety if the workforce lacks the specific skills to use it to its full potential. Locally, Malak et al. (2022) identified a strong need to improve patient safety culture among emergency nurses in Jordan. Regionally, studies in Palestinian hospitals (Salameh et al., 2023) and broader evaluations of clinical decision-making (Abu Arra et al., 2023) further underscore how workforce factors and the clinical environment dictate nursing competency and safety outcomes.

To the best of our knowledge, no current study in Jordan evaluates the direct association between DHL and perceptions of PSC among nurses, nor does one compare DHL levels across key demographic and professional subgroups. Addressing these gaps is crucial for optimising the benefits of digital health technologies in Jordanian healthcare settings. Therefore, the primary aim of this multi-centre study was to investigate the relationships between digital health literacy (DHL), patient safety culture (PSC), clinical decision-making, and technology acceptance among registered nurses in Jordan. By identifying how DHL relates to safety culture and how it varies across nursing groups, the findings will provide the evidence needed to strengthen digital capacity, guide targeted professional development, and ultimately contribute to building a digitally competent nursing workforce that enhances patient safety and benefits the wider community.

Based on the theoretical framework and the existing literature, the following hypothesis was formulated: There is a significant positive association between Jordanian nurses’ DHL and their perceptions of PSC.

## Methods

### Study design

A multi-centre, cross-sectional survey design was employed to investigate the association between DHL and PSC among registered nurses in Jordan. This design was chosen for its suitability in concurrently assessing multiple constructs and exploring relationships within a large, diverse sample at a single point in time. The data collection phase was completed in the three-month period from July to September 2025.

### Setting and participants

The study was conducted across five major hospitals in Jordan, purposefully selected to ensure a diverse representation of the national healthcare landscape. The settings include two Ministry of Health (public) facilities, one university-affiliated teaching hospital, and two private hospitals. This diverse sampling frame allowed for a comprehensive analysis of the nursing workforce across different healthcare delivery models.

The target population consisted of registered nurses engaged in direct patient care. Eligible participants must meet the following inclusion criteria: (1) be a registered nurse, (2) be currently employed in a role involving direct patient care, and (3) have a minimum of six months of clinical experience at their respective institution. Nurses in purely administrative roles and those on extended leave (e.g. maternity or long-term sick leave) during the data collection period were excluded from the study.

### Sampling strategy and sample size

A two-stage sampling strategy was employed was employed to enhance the representativeness of the sample and to minimise potential sampling bias. The total population of eligible nurses across the five hospitals served as the sampling frame. First, the frame was conducted purposively to capture a diverse cross-section of hospital types (public, private, university-affiliated), clinical unit: (ICU, medical-surgical, emergency) and professional role: (staff, charge, managerial). Second, within these selected types, participants were randomly selected based on the number of nurses drawn from each type being proportional to its size in the overall sampling frame. This method ensured that the final sample accurately reflected the diversity of the nursing workforce in Jordan and guaranteed sufficient subgroup sizes for robust comparative analysis.

The required sample size was determined based on the guidelines for Structural Equation Modeling (SEM), which was the primary statistical method for this study. According to established standards, SEM requires at least 10–20 participants per estimated parameter to ensure model stability and produce reliable results (Kline, 2016).

The proposed theoretical model for this study included 25 estimated parameters, covering the factor loadings, error variances, and the structural path between the latent variables of DHL and PSC. Missing data were handled using full information maximum likelihood in Mplus, which utilises all available data points to estimate model parameters without excluding participants. Applying the more conservative ratio of 20 participants per parameter, a baseline sample of at least 500 nurses was deemed necessary.

### Data collection instrument

A structured, self-administered questionnaire was developed for this study following a comprehensive review of the relevant literature and established theoretical frameworks (Jenkins, 1985; Kuek & Hakkennes, 2020; Sexton et al., 2006; Ting et al., 2021; van der Vaart & Drossaert, 2017). The resulting instrument was a composite tool composed of seven sections. Key sections were adapted from previously validated and widely used scales to ensure content and construct validity, with items tailored to the context of nursing practice in Jordan. Adaptation steps included no translation (kept in English) and adjusting terminology for the nursing context. Content validity for these specifically adapted items was evaluated and approved by the expert panel prior to use.

- Section 1: Demographics. This section collected data on participant age, gender, years of nursing experience, highest educational qualification, current professional role, primary clinical unit, hospital type, and average weekly working hours.
- Section 2: Digital Health Literacy Instrument (DHLI). Nurses’ DHL were assessed using a 24-item nurse-adapted version of the DHLI. This instrument measures 6 domains on a 4-point Likert scale from 1 (*Very difficult*) to 4 (*Very easy*).
- Section 3: Patient Safety Culture (PSC). Perceptions of patient safety culture were measured using a 12-item version of the safety attitudes questionnaire (SAQ), which evaluated key dimensions on a 5-point Likert scale from 1 (*Strongly disagree*) to 5 (*Strongly agree*).
- Section 4: Clinical Decision-Making in Nursing Scale (CDMNS). The 8-item CDMNS was used to assess nurses’ decision-making processes on a 5-point Likert scale from 1 (*Never*) to 5 (*Always*).
- Section 5: Technology Acceptance (TA). This 6-item section, based on the Technology Acceptance Model (TAM), measured Perceived Usefulness, Perceived Ease of Use, and Behavioral Intention on a 5-point Likert scale from 1 (*Strongly disagree*) to 5 (*Strongly agree*).
- Section 6: Attitudes Toward Technology (ATT). A 4-item scale assessed general attitudes, trust, and confidence related to digital health technologies on a 5-point Likert scale.
- Section 7: Barriers and Facilities (B&F). This 8-item section identified perceived organizational barriers and facilitators to the use of digital health tools, rated on a 5-point scale from 1 (*Not a barrier/facilitator*) to 5 (*Major barrier/facilitator*).

### Instrument validity and reliability

The instruments selected for this study (DHLI, PSC, CDMNS, TA, ATT and B&F) have been widely used and demonstrate strong psychometric properties in previous research (Sexton et al., 2006; Ting et al., 2021; van der Vaart & Drossaert, 2017). Content validity of the overall questionnaire was established through a review by a multidisciplinary expert panel consisting of two senior nurse researchers, one clinical informatics specialist, and two senior nursing managers.

A pilot study was conducted with 15 registered nurses (not included in the main sample) to assess the clarity, comprehensibility, and face validity of the questionnaire. Feedback from the pilot study was used to make final refinements. Internal consistency reliability was assessed for the pilot and main study data by calculating Cronbach's alpha for each scale and subscale. A Cronbach's alpha coefficient of ≥ 0.70 was considered acceptable.

### Data collection procedure

Data collection was conducted in two phases. Phase one, the preparatory stage, involved securing ethical approvals and training research assistants in standardised procedures for recruitment, informed consent, and survey administration to ensure data quality and minimise bias.

In phase two, the survey administration phase, trained research assistants approached eligible nurses during various shifts. To maximise participation, a dual-mode approach combining paper-based and secure online questionnaires was offered. Written informed consent was obtained from each participant prior to their involvement. To increase the response rate, scheduled reminders were sent to non-respondents at two-week intervals.

In total, 800 nurses were approached, of which 714 were deemed eligible based on the inclusion criteria. A final sample of 500 completed the survey, yielding a response rate of 70%

### Ethical considerations

Ethical approval for this study was obtained from the University of Petra Research Ethics Committee (Approval No: S/16/8/2025).

### Statistical analysis

All data were coded and analyzed using IBM SPSS Statistics version 29 and Mplus version 8.0 for the SEM analysis.

- Descriptive Statistics: Frequencies and percentages were used for categorical data, while means and standard deviations were calculated for continuous variables.
- Normality Testing: The normality of distribution for continuous variables were assessed using the Shapiro–Wilk test. As some data were anticipated to be non-normally distributed (*p* < 0.05), appropriate non-parametric tests were employed for certain inferential analyses.
- Inferential Statistics:
- Chi-square tests were used for associations between categorical variables.
  o For comparing DHL scores across subgroups, Independent t-tests (or the Mann–Whitney U test for non-normal data) were used for binary groups, and one-way ANOVA (or the Kruskal–Wallis test for non-normal data) were used for groups with more than two categories. For significant Kruskal–Wallis results, post-hoc pairwise comparisons were conducted using Dunn's test with a Bonferroni correction for multiple testing.
  o The primary hypothesis was tested using SEM. The model fit was evaluated using established goodness-of-fit (GoF) indices and thresholds: the Comparative Fit Index (CFI) and Tucker-Lewis Index (TLI) ≥ 0.95 for excellent fit and ≥ 0.90 for acceptable fit, the Root Mean Square Error of Approximation (RMSEA) ≤ 0.06, and the Standardised Root Mean Square Residual (SRMR) ≤ 0.08 (Hu & Bentler, 1999; Kline, 2016).

For all inferential tests, a two-tailed *p*-value ≤ 0.05 was considered statistically significant.

## Results

### Participant characteristics

A total of 500 registered nurses completed the survey. The demographic and professional characteristics of the sample are detailed in Table 1. The majority of respondents were female (81.0%, n = 405), with a mean age of 33.1 years (SD = 7.5). The average length of nursing experience was 10.2 years (SD = 6.9), and the average reported work week was 42.5 h (SD = 5.8).

**Table 1.** Demographic and professional characteristics of participants (N = 500)

Characteristic	Category	*N* (%)
Age Group (years)	21–29	205 (41)
	30–39	151 (30.2)
	40–49	95 (19.0)
	50+	49 (9.8)
Gender	Female	405 (81.0)
	Male	95 (19.0)
Years of Experience	<1 year	76 (15.2)
	1–5 years	174 (34.8)
	6–10 years	129 (25.8)
	10 + years	121 (24.2)
Education Level	Diploma	100 (20.0)
	BSc	350 (70.0)
	MSc	38 (7.6)
	PhD	12 (2.4)
Current Role	Staff Nurse	375 (75.0)
	Charge Nurse	75 (15.0)
	Nurse Manager	50 (10.0)
Clinical Unit	Medical-Surgical	200 (40.0)
	ICU	100 (20.0)
	Emergency	100 (20.0)
	Outpatient	75 (15.0)
	Other	25 (5.0)
Hospital Type	Public	210 (42.0)
	Private	165 (33.0)
	University-affiliated	125 (25.0)
Average Weekly Hours	< 40	133 (26.6)
	40–49	279 (55.8)
	≥ 50	88 (17.6)

The most common educational qualification was a Bachelor of Science in Nursing (70.0%, n = 350), and the most frequently reported clinical unit was Medical-Surgical (40.0%, n = 200). Participants were predominantly staff nurses (75.0%, n = 375) and were distributed across public (42.0%, n = 210), private (33.0%, n = 165), and university-affiliated (25.0%, n = 125) hospitals, ensuring a representative sample from diverse clinical settings.

### Instrument reliability

Internal consistency reliability was assessed for all scales and subscales using Cronbach's alpha. As shown in Table 2, all instruments demonstrated acceptable to excellent reliability. The overall scales for the primary constructs showed high internal consistency: DHLI (α = 0.92), PSC (α = 0.94), CDMNS (α = 0.91), and TA (α = 0.93). All subscales exceeded the acceptable threshold of α ≥ 0.70, confirming that the instruments provided reliable measurements for the main study data.

**Table 2.** Internal consistency reliability of study instruments (N = 500).

Construct	Subscale	Number of Items	Cronbach's Alpha (α)
DHLI	Overall	24	0.92
	Operational Skills	4	0.85
	Navigation Skills	4	0.83
	Information Searching	4	0.81
	Evaluating Reliability	4	0.78
	Determining Relevance	4	0.79
	Protecting Privacy	4	0.75
PSC	Overall	12	0.94
	Teamwork Climate	2	0.88
	Safety Climate	2	0.85
	Job Satisfaction	2	0.84
	Stress Recognition	2	0.77
	Perceptions of Management	2	0.89
	Working Conditions	2	0.82
CDMNS	Overall	8	0.91
	Search for Alternatives	2	0.92
	Canvassing Objectives	2	0.88
	Evaluation of Consequences	2	0.94
	Search for Information	2	0.89
TA	Overall	6	0.93
	Perceived Usefulness	2	0.88
	Perceived Ease of Use	2	0.86
	Behavioural Intention / Adoption	2	0.84
ATT	Overall	4	0.78
B&F	Barriers Subscale	4	0.79
	Facilitators Subscale	4	0.81

### Descriptive statistics for key constructs

The overall mean scores for the primary constructs and their respective subscales are presented in Table 3. Nurses reported a moderately high level of DHL (M = 3.28, SD = 0.58). The highest-rated DHLI subscale was operational skills (M = 3.65), while the lowest was evaluating reliability (M = 2.91). Perceptions of PSC were positive overall (M = 3.71, SD = 0.68), with Teamwork Climate receiving the highest score (M = 4.21) and perceptions of management the lowest (M = 3.34). Technology acceptance was strong, with perceived usefulness (M = 4.27) rated more highly than perceived ease of use (M = 3.83).

**Table 3.** Descriptive statistics of key study constructs.

Construct	Subscale	Mean (SD)	Scale Range
DHLI		3.28 (0.58)	1–4
	Operational Skills	3.65 (0.45)	
	Navigation Skills	3.54 (0.52)	
	Information Searching	3.21 (0.68)	
	Evaluating Reliability	2.91 (0.74)	
	Determining Relevance	3.12 (0.65)	
	Protecting Privacy	3.25 (0.70)	
PSC		3.71 (0.65)	1–5
	Teamwork Climate	4.21 (0.72)	
	Safety Climate	3.80 (0.81)	
	Job Satisfaction	3.70 (0.95)	
	Stress Recognition	3.55 (1.05)	
	Perceptions of Management	3.34 (0.92)	
	Working Conditions	3.65 (0.88)	
CDMNS		3.95 (0.61)	
	Search for Alternatives	3.75 (0.75)	
	Canvassing Objectives	4.05 (0.70)	
	Evaluation of Consequences	3.90 (0.72)	
	Search for Information	4.10 (0.65)	
TA	Perceived Usefulness	4.27 (0.75)	
	Perceived Ease of Use	3.83 (0.90)	
	Behavioural Intention / Adoption	3.29 (0.84)	
ATT		4.08 (0.75)	
B&F	Barriers	3.61 (1.04)	
	Facilitators	3.84 (0.88)	

### Association between digital health literacy and patient safety culture

To test the primary hypothesis, a SEM was specified to assess the predictive relationship between the latent constructs of DHL and PSC. The model's goodness-of-fit indices are presented in Table 4. All indices met or exceeded the recommended thresholds for an excellent fit, indicating that the theoretical model accurately represented the relationships observed in the sample data.

**Table 4.** Goodness-of-fit indices for the structural equation model (N = 500).

Fit Index	Value	Interpretation
Chi-square ratio (χ²/df)	2.53	Excellent
CFI	0.95	Excellent
TLI	0.94	Acceptable
RMSEA	0.056	Excellent
SRMR	0.051	Excellent

The results of the structural path analysis are presented in Table 5. A statistically significant and strong positive path was found from DHL to PSC. This finding supported the primary hypothesis of the study. The model successfully explained 28.1% of the variance (R²) in PSC, indicating a substantial predictive relationship.

**Table 5.** Standardised path coefficients for the structural model predicting patient safety culture.

Path	Standardised Coefficient (β)	Standard Error (SE)	95% CI	*p*-value	R²
Digital Health Literacy → Patient Safety Culture	0.42	0.05	[0.32, 0.52]	**<.001**	0.281

Bolded values are statistically significant at p < 0.05.

Note: SEM parameter estimates and fit indices are sensitive to sample size; however, our robust sample of 500 adequately powered the detection of this structural path.

### Comparison of digital health literacy levels across subgroups

To explore comprehensively the influence of demographic and professional variables on DHL, a series of independent samples t-tests and ANOVA were conducted. The results for all variables are detailed in Table 6.

**Table 6.** Comparison of mean DHLI scores across All demographic and professional subgroups (N = 500).

Group	Category	Test Statistic	*p*-value
Age Group	21–29	F = 0.89	0.445
	30–39		
	40–49		
	50+		
Gender	Female	t = 0.98	0.327
	Male		
Years of Experience	<1 year	F = 0.26	0.851
	1–5 years		
	6–10 years		
	10 + years		
Education Level	Diploma	F = 9.21	**<0**.**001**
	BSc		
	MSc		
	PhD		
Current Role	Staff Nurse	F = 1.67	0.189
	Charge Nurse		
	Nurse Manager		
Clinical Unit	Medical-Surgical	F = 4.15	**0**.**008**
	ICU		
	Emergency		
	Outpatient		
	Other		
Hospital Type	Public	F = 6.88	**0**.**001**
	Private		
	University-affiliated		
Average Weekly Hours	<40	F = 0.45	0.638
	40–49		
	≥50		

Bolded values are statistically significant at *p* < 0.05.

The analysis revealed that DHL levels varied significantly across several key groups. Statistically significant differences were found for education level, hospital type, and clinical unit. Post-hoc analysis indicated that nurses with higher academic qualifications consistently reported higher DHL. Similarly, nurses working in university-affiliated hospitals demonstrated significantly higher DHL scores compared to their peers in public hospitals. Among clinical units, ICU nurses reported the highest mean DHL scores.

In contrast, no statistically significant differences were found in mean DHL scores based on gender, current role, years of nursing experience, age group, or average weekly hours. These findings suggested that while organizational context and educational background were strong correlates of digital literacy, factors related to individual experience and workload might be less influential in this sample.

## Discussion

The aim of this multi-centre study was to investigate the relationship between DHL and perceptions of PSC among registered nurses in Jordan. A secondary objective was to identify the demographic and professional factors associated with varying levels of DHL. The study's findings confirmed a significant, positive, and robust association between these two core constructs, suggesting that an individual nurse's digital competency was a critical component in fostering a broader organisational culture of safety. Furthermore, the results highlighted key disparities in DHL, with education level and hospital type emerging as significant factors. Nurses demonstrated a strong acceptance of digital technologies but identified significant organisational barriers, primarily a lack of adequate training and time.

This study had several strengths that bolstered the credibility and significance of its findings. A primary strength of this study was its large and robust sample size. This sample far exceeded the minimum requirements for conducting a stable and reliable SEM analysis, ensuring high statistical power and reducing the risk of a Type II error (failing to detect a true effect). This large sample also provided a solid foundation for the credibility of the subgroup analyses. Furthermore, the use of a multi-centre, stratified random sampling strategy across public, private, and university-affiliated hospitals was a significant methodological strength. This approach minimised selection bias and enhanced the representativeness of the sample, allowing the findings to be more generalisable to the broader nursing workforce in Jordan than a single-site or convenience-based sample would permit. The study's internal validity was bolstered by the use of well-established and validated instruments, including the PSC and the DHLI. The use of these standardised tools ensured that the constructs of PSC and DHL were measured with a high degree of reliability and validity. The comprehensive nature of the questionnaire, which also captured data on technology acceptance, clinical decision-making, and organisational barriers, allowed for a holistic and nuanced interpretation of the central findings.

The central finding of this study, a direct, positive predictive relationship between nurses’ DHL and their perception of PSC (β = 0.42), established a novel and critical link in the literature. While previous research in Jordan by Al-Jarrah (2024) demonstrated that the presence of Digital Healthcare Technologies (DHT) impacts patient safety culture, our study added a crucial layer by focusing on the human factor. We showed that it was not merely the availability of technology, but the nurse's individual competency in using that technology that served as a significant predictor of a safer care environment. Higher DHL directly translates to greater efficiency and confidence in using essential clinical systems. When nurses can navigate systems smoothly (as reflected in high scores for Operational Skills), they may experience less cognitive load and frustration. Although speculative since it was not directly measured, this may allow them to dedicate more mental resources to safety-critical tasks (Kuek & Hakkennes, 2020; Thomas, 2023). This refines the conclusions of studies, such as Wurster et al. (2024), which found that EHRimplementation can lead to inconsistent changes in documentation; our results suggested that higher DHL might be a key factor in maximising the benefits while mitigating the risks.

Our descriptive findings on the components of DHL were remarkably consistent with the Jordanian study by Shudayfat et al. (2023), who also found ‘very desirable results' in operational skills but lower proficiency in more complex domains. Our study specified this gap in the domain of Evaluating Reliability (M = 2.91), suggesting a persistent trend where nurses were well-trained on procedural system use but received less support in the higher-order cognitive skills needed for evidence-based practice. This distinction is central to the design of the DHLI (van der Vaart & Drossaert, 2017) and is particularly concerning in light of the systematic review by Hants et al. (2023), which found that clinical reasoning is often inadequately captured by digital health systems. Nurses with lower skills in evaluating information might be less equipped to compensate for these system-level deficiencies, potentially impacting patient safety (Hants et al., 2023; van der Vaart & Drossaert, 2017). To explicitly address this weakness in evaluating reliability, healthcare organisations should design concrete interventions, such as specialised evidence-appraisal training modules, digital checklists embedded directly into clinical workflows, and enhanced clinical decision support systems.

Our analysis of demographic factors provided a nuanced perspective on the ‘digital divide,' pointing to systemic rather than generational factors. The finding that higher Education Level was significantly associated with higher DHL contrasted with Abed et al. (2022), who found no link between education and nurses’ attitudes toward EHRs. This suggests a critical distinction: while formal education might not change a nurse's general attitude, it appeared highly effective at building the specific skills that constitute DHL.

More telling was the finding related to Hospital Type, where university-affiliated hospitals showed significantly higher DHL. These institutions, as hubs for education and research, likely foster a culture of continuous learning and invest more heavily in structured training, suggesting that the organisational environment was a powerful driver of nurse competency. Importantly, our result that DHL did not significantly vary by age or years of experience confirmed the findings of Abed et al. (2022) but contrasted with some Western literature where older staff reported lower confidence (Kuek & Hakkennes, 2020). This may indicate that in the Jordanian context, widespread exposure to national EHR systems has levelled the playing field, making institutional training and system quality more determinant of skill than age.

This study also expanded the assessment of PSC to include Job Satisfaction and Stress Recognition, providing a more holistic view. The moderately positive score for Job Satisfaction (M = 3.70) alongside lower scores for Stress Recognition (M = 3.55) suggested that while nurses might like their roles, they acknowledged that performance was impaired by stressors, such as excessive workload and fatigue. This finding directly supported the literature on nurse burnout, where administrative burdens from digital systems are identified as a major contributor to stress (Alnasser et al., 2024; Thomas, 2023). The recognition that tense situations increased the likelihood of errors was particularly critical, as it provided a direct perceptual link between the work environment and patient safety outcomes. This aligned with the work of Kuek and Hakkennes (2020), who identified anxiety about technology use as a key issue to be addressed in information systems implementation.

The perceptions of PSC in our sample aligned with regional trends. High scores for Teamwork Climate and lower scores for Perceptions of Management mirrored findings from Lebanon (El-Jardali et al., 2011) and the original PSC validation studies (Sexton et al., 2006). This suggests that while frontline collaboration was a strength, systemic issues related to administrative support remained a challenge. The low score for Perceptions of Management might also explain why ‘lack of training' and ‘insufficient time' were the top-rated barriers. A comprehensive integrative review by Ting et al. (2021) identified inadequate training as a primary impediment to EHR implementation, while the issue of time constraints and workload was a direct echo of findings on nurse burnout (Alnasser et al., 2024; Thomas, 2023). The facilitators identified, such as management support, were likewise recognised as critical success factors for technology adoption, underscoring the pivotal role the organisation plays in enabling the effective and safe use of digital tools (Ibrahim et al., 2024; Kuek & Hakkennes, 2020).

## Limitations

Despite these strengths, several limitations must be acknowledged. First, the cross-sectional design of the study allowed for the identification of associations but precluded the establishment of causality. While we found that higher DHL predicted a more positive PSC, it was also plausible that a strong safety culture (which may include better training and support) fostered higher DHL in nurses. A longitudinal study would be required to explore the causal direction of this relationship over time. Second, the reliance on self-reported data for all constructs introduced the potential for social desirability bias. Nurses might have over-reported their digital skills (DHL) or provided more favourable assessments of their workplace's safety culture (PSC) than an objective assessment would reveal. While the assurance of anonymity was intended to mitigate this risk, future research could incorporate objective measures, such as performance-based digital literacy tasks or direct observation of safety practices, to complement these perceptual findings. Third, while the multi-centre design enhanced generalizability within the Jordanian context, the findings might not be directly transferable to other countries. The specific healthcare systems, national digital health strategies, and nursing education standards in Jordan create a unique environment that shapes the relationship between DHL and PSC. Therefore, caution should be exercised when applying these results to different cultural or healthcare contexts. Furthermore, the dual-mode data collection approach (paper vs. online) may have introduced mode effects. There is also a risk of selection bias concerning which nurses were available and consented to participate during the data collection shifts. Finally, unmeasured confounders, such as prior formal informatics training or overall tenure using EHR systems, were not accounted for in our models. Finally, because all data were collected from a single source at a single point in time, there is a possibility of common method variance (CMV), which could potentially inflate the strength of the observed relationships between variables. Although this is an inherent limitation in cross-sectional survey research, the study's robust theoretical framework and use of multi-item, validated scales helped to minimise this concern.

## Conclusions

In conclusion, this multi-centre study provided compelling evidence that DHL was a significant and positive predictor of PSC among registered nurses in Jordan. The findings established that an individual nurse’s competency with digital health technologies was not merely a technical skill but a foundational component of a safer healthcare environment. This research successfully bridged a critical gap by demonstrating that while the presence of digital systems was important, it was the human factor, the nurse's ability to effectively find, appraise, and apply digital information, that was strongly associated with a positive culture of safety. Furthermore, interventions must also address the significant impact of workload and stress on performance by optimising digital workflows, which might in turn improve both patient safety and job satisfaction. Given the unique health policy and nursing education context in Jordan, these findings should be interpreted with caution. Explicit cross-country validation is recommended to determine the transferability of these structural relationships to other international healthcare systems.

The study also revealed that key organisational and systemic factors, such as the nurse's level of education and the type of hospital they work in, were more influential drivers of DHL than individual demographic characteristics, such as age or years of experience. This challenged the common stereotype of a generational digital divide and instead pointed toward the critical role of organisational investment in training, supportive digital infrastructures, and fostering a culture of continuous learning.

While this study established a strong association, its cross-sectional design did not allow for causal inference. Future longitudinal research is recommended to track how changes in DHL over time, perhaps following a targeted educational intervention, impact PSC. Additionally, future studies could employ objective measures of safety (e.g. error reporting rates) to complement the perceptual data gathered here. Ultimately, this research underscored that to fully realise the promise of technology to improve patient outcomes, healthcare institutions must invest as much in the digital competency of their nurses as they do in the digital systems themselves.

## Supplementary Material

Supplementary File 1 Data.xlsx

## Data Availability

All data analysed during this study are included in this published article and its Supplemental Material.
